# Morpho-Functional and Biochemical Characterization of Adrenal Masses in a Heterogeneous Cancer Population

**DOI:** 10.3390/cancers17172897

**Published:** 2025-09-03

**Authors:** Rosaria Meucci, Antonio Stigliano, Giuseppe Campagna, Francesco Garaci, Tommaso Pintus, Guido Roberto, Alberto Signore

**Affiliations:** 1 U.O.C. Diagnostic Imaging, PTV Policlinico “Tor Vergata” University, Viale Oxford 81, 00133 Rome, Italy; francesco.garaci@ptvonline.it; 2 Nuclear Medicine Unit, Sant’Andrea University Hospital of Rome, Via di Grottarossa 1035, 00189 Rome, Italy; gius.campagna@gmail.com (G.C.); tommaso.pintus@gmail.com (T.P.); alberto.signore@uniroma1.it (A.S.); 3 Endocrinology Unit, Sant’Andrea University Hospital of Rome, Via di Grottarossa 1035, 00189 Rome, Italy; antonio.stigliano@uniroma1.it (A.S.); guido.roberto@uniroma1.it (G.R.); 4 Department of Clinical and Molecular Medicine, “Sapienza” University of Rome, Sant’Andrea University Hospital, 00161 Rome, Italy

**Keywords:** adrenal mass, cancer therapy, secreting adenomas, non-secreting adenomas, computed tomography, positron emission tomography

## Abstract

**Background/Objectives**: The aim of this retrospective study is to analyze the prevalence of adrenal masses in patients with different types of tumors who performed [^18^F]FDG PET/CT. **Method**: Eighty-eight patients who performed [^18^F]FDG PET/CT from 2019 to 2024 for oncologic purposes, with evidence of adrenal mass and different types of cancer, were retrospectively retrieved from our database. To assess the functionality of the adrenal lesions, ACTH, adrenal steroids, and a low-dose dexamethasone suppression test (DST-1 mg) was performed in the Endocrinology Unit. According to the results of DST-1 mg, the adrenal lesions were classified in cortisol-secreting lesions (CS) and non-secreting lesions (NS). **Results**: Adrenal masses were most prevalent in breast cancer (25/88, 28.4%), followed by kidney cancer (13/88, 14.8%), thyroid cancer (11/88, 12.5%), and the others. We report a global rate of 39.8% (34/88) cortisol-secreting amongst all adrenal lesions. **Conclusions**: Our study is the largest retrospective study available in the literature calculating the prevalence of adrenal lesions in several cancer patients. From our results there emerges a high prevalence of secreting lesions. Therefore, biochemical characterization of adrenal masses is crucial in oncological settings to address clinical and therapeutic management.

## Simple Summary

The aim of this retrospective study is to analyze the prevalence of adrenal masses in paients with different types of tumors who performed [^18^F]FDG PET/CT. We analyze 88 patients who performed [^18^F]FDG PET/CT from 2019 to 2024 for oncologic purposes, with evidence of adrenal mass and different types of cancer, which were retrospectively retrieved from our database. We report the prevalences of the different type of cancers. To assess the functionality of the adrenal lesions of all patients who underwent hormonal tests; we found a global rate of 39.8% (34/88) cortisol secreting amongst all adrenal lesions. Our study is the largest retrospective study available in the literature calculating the prevalence of adrenal lesions in several cancer patients. From our results there emerges a high prevalence of secreting lesions. Therefore, biochemical characterization of adrenal masses is crucial in oncological settings to address clinical and therapeutic management.

## 1. Introduction

The discovery of adrenal masses during radio imaging, such as computed tomography (CT), magnetic resonance (MR), and ^18^Fluorine-Fluorodeoxyglucose [^18^F]FDG positron emission tomography/computed tomography (PET/CT), is a frequent occurrence.

According to autoptic and radiological studies, the prevalence in the general population of an adrenal mass incidentally discovered varies between 1.4% and 8.7%, particularly in the elderly [1,2]. Adrenal incidentalomas incidence showed a 10-fold increase from 1995 to 2017, and it is still growing together with the increasing number of diagnostic examinations being performed [3,4,5].

Beyond the definition of incidentaloma, an adrenal mass, discovered by imaging for tumor staging or follow-up of a recognized neoplasia, does not fall within the definition of incidentaloma [6].

Adrenal masses usually require a complete diagnostic assessment aiming at differentiating benign and malignant lesions; a state-of-the-art imaging assessment does not allow for differentiating between secreting and non-secreting adenomas for which biochemical markers are required; both imaging features and biochemical parameters play an essential role in the differential diagnosis of adrenal lesions. Among the benign masses with a different etiology, non-secreting adenomas represent 70% of adrenal masses.

The prevalence of adrenal malignant lesions, including primary neoplasia and metastasis, ranges between 5% and 10% [6,7]. Both imaging features and biochemical parameters play an essential role in the differential diagnosis of adrenal lesions [6,7,8,9]. Recent data in the literature including the clinical practice guidelines of the European Society of Endocrinology (ESE) and European Network of Study of Adrenal Tumors (ENSAT) agree that every patient with the incidental finding of an adrenal mass should undergo a clinical assessment, diagnostic path, and imaging studies to determine the nature of the adrenal lesion and hormonal work-up to investigate whether the adenoma is associated with hormonal overt secretion or not [6,10,11,12,13,14,15].

The evidence of adrenal lesions in patients with a previous oncological history has been traced back in epidemiological estimates with a high frequency to metastatic localization of the adrenal gland in a particularly high percentage: 50–75% [16,17]. This consideration in previous years has been attributed to, in many cases, through diagnostic imaging, the presumed condition of disease progression [18,19].

Radiologic imaging modalities, CTs and MRIs, are crucial to distinguish between benign and malignant lesions. A no-contrast-enhanced CT allows for the identification of “lipid-rich” adenomas through Hounsfield Units (HU) < than 10; values higher than 10 HU need further investigations to distinguish benign from malignant lesions. Chemical shift MR imaging may enable characterization of additional adenomas when results of CT densitometry are indeterminate [20]. Other studies have shown that for lipid-rich adenomas, there is no significant difference in differentiating adrenal adenomas from malignant adrenal masses between a chemical shift MRI and unenhanced CT densitometry, but a chemical shift MRI might be superior when evaluating lipid-poor adenomas with an attenuation value up to 30 HU on a CT scan [11,21].

Moreover, a contrast-enhanced CT improves the characterization of adrenal lesions. Blake et al., in their study, determined that, with a 10-min-delayed multidetector row CT protocol, the attenuation and wash-out criteria are clarified, which help optimize the differentiation between benign and malignant adrenal masses [21].

In addition to the CTs and MRIs mentioned above, [^18^F]FDG PET/CT plays a leading role in the functional and pathological interpretation of an adrenal lesion; the metabolic features of these findings allow for a non-invasive assessment [22,23]. It is broadly accepted that [^18^F]FDG PET/CT is highly accurate in differentiating benign from malignant adrenal masses. A study reported that [^18^F]FDG PET/CT shows a 100% sensitivity and 93.8% specificity [21]; other studies support these data [24]. A systematic review and meta-analysis showed that most adrenal masses can be characterized using [^18^F]FDG PET/CT with a high sensitivity (97%), specificity (91%), and accuracy, with an area under the curve (AUC) of 0.98 [25].

Furthermore, it has been observed that different degrees of radiopharmaceutical uptake, expressed in terms of a standardized uptake value (SUV), correspond to different hormonal behaviors, with maximum uptake values in cortisol-secreting adenomas (SUV max: 10.1 ± 3.2) [26]. Adrenal metastasis localizations show a high SUVmax as primary cancer lesions; indeed, benign adrenal masses discovered accidentally (either functioning or non-functioning adenomas) generally show a SUVmax < 3 [26,27].

An accurate characterization of adrenal masses is even more crucial in cancer patients in which the exclusion of the repetitive nature of the adrenal lesion is of paramount importance. In the case of adenomas, a complete endocrinological assessment is mandatory to perform a correct diagnosis, so promptly treating symptoms of hormonal excess, which may further complicate the management of cancer patients, is important. In the last few years, growing interest has arisen in properly identifying secreting adrenal adenomas; hyper-aldosteronism is the leading cause of secondary hypertension, and adrenal incidentalomas have a prevalence in the general population of around 1–6% [27]. Based on these considerations, the frequent finding of adrenal masses in cancer patients requires an appropriate differential diagnosis between cancer metastasis localizations and other benign or primary malignant lesions.

Starting from this evidence, the aim of this retrospective study is to analyze the prevalence of adrenal masses with different secretion patterns in patients with several types of neoplasia who performed [^18^F]FDG PET/CT.

## 2. Patients and Methods

### 2.1. Inclusion Criteria

The present study included all patients who performed [^18^F]FDG PET/CT from 2019 to 2024 for oncological follow-up in which we found an adrenal lesion on the CT scan and that performed a low-dose dexamethasone suppression test of 1 mg (DST-1 mg).

Patients had different types of cancers of different stages.

All patients had a histological diagnosis of cancer and were clinically managed at the standard of care in the Oncologic Unit. The study was conducted in accordance with the Declaration of Helsinki. Patients enrolled in this study have given consent for their data to be presented in anonymized form.

### 2.2. Homonal Evaluation

To assess the functionality of the adrenal mass, patients performed, in the Endocrinology Unit, the following assays: ACTH, Cortisol, Androstenedione, 17 hydroxyprogesterone, Testosterone, Dehydroepiandrosterone sulphate, 24 h urinary metanephrines, and aldosterone and plasma renin activity (only in hypertensive patients); on another day, they performed a low-dose dexamethasone suppression test of 1 mg (DST-1 mg), administered orally between 11 PM and midnight, and cortisol levels are drawn the next morning between 8 am and 9 am. The test is made in the presence of unequivocal positivity to at least three screening tests and after the exclusion of some disorders, such as major depression, chronic alcoholism, metabolic syndrome, and polycystic ovary syndrome (pseudo-Cushing’s state) [8]. The following conditions have been excluded: (i) drug interactions interfering with dexamethasone metabolism; (ii) wrong dexamethasone intake or failure to respect the protocol; (iii) alteration of feedback mechanisms in elderly patients or those with comorbidities; (iv) use of estro-progestins leading to elevated cortisol-binding globulin levels; and (v) liver or kidney impairment. Steroids were assayed by a chemiluminescent microparticle immunoassay (Alinity Instrument, Abbott, Chicago, IL, USA), while plasma ACTH levels were determined using a chemiluminescent immunoassay (CLIA) (Liaison XL analyzer, DiaSorin, Saluggia, Italy). Patients with falling ≤49 mmol/L at DST-1 mg were considered to have a non-secreting pattern; on the contrary, cortisol levels above 50 mmol/L over time (at least in triplicate) were considered to have “autonomous cortisol secretion” due to a secreting lesion [8]. According to current guidelines, through the DST-1 mg, the other test mentioned above, and due to a multidisciplinary team approach, the patients were classified with cortisol-secreting (CS) and non-secreting lesions (NS).

### 2.3. Imaging Analyses

PET/TC allows for non-invasive quantitative assessments of functional and biochemical processes. Whole-body PET/CT scans were acquired with a dedicated hybrid PET/CT Biograph (Siemens, Munich, Germany) 50–60 min post intra-venous [^18^F]FDG injection. After a scout CT, for the definition of the field of study, a low-dose CT scan (120 mA), without contrast, was acquired for attenuation correction, according to current guidelines for [^18^F]FDG PET/CT imaging in cancer. CT scan is performed only for attenuation correction (CT-AC) and anatomical correlation of PET findings (with reduced voltage and/or current of the X-ray tube settings), i.e., a low-dose CT is not intended for dedicated radiological interpretation [28].

### 2.4. Statistical Analysis

Continuous variables were presented as mean ± standard deviation (SD) and categorical variables were shown as absolute frequency and percentage, *n* (%).

Descriptive data were calculated using specific procedures written in SAS language (“proc means” and “proc freq”, for continuous and categorical variables, respectively).

Statistical analysis was performed using SAS version 9.4 TS Level 1 M8 and JMP PRO version 17 (SAS Institute, Cary, NC, USA).

## 3. Results

The study included a total of 88 oncological patients with adrenal lesions; the mean age was 66.4 ± 9.6 years, including 30 men (34.1%, mean age: 68.5 ± 11.7 years) and 58 women (65.9%, mean age: 65.4 ± 8.1 years). The adrenal lesion diameters ranged between 10 and 26 mm (average 22.3 mm).

DST-1 mg identified 53 NS (60.2%) and 35 CS (39.8%).

All adrenal lesions studied in our population did not change in size during the entire follow-up period (5 years). Furthermore, they did not show a pathological uptake on PET [^18^F]FDG with a SUVmax value <3.

**Figure 1:**
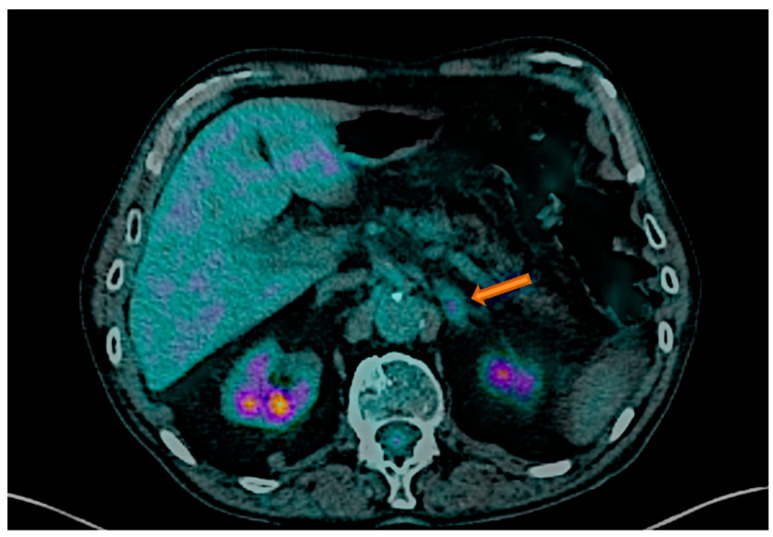
[^18^F]FDG PET/CT of 59-year-old female with history of breast cancer. Left low-density adrenal secreting-lesion indicated by the arrow (SUV max: 2.59; UH −10.65).

**Figure 2:**
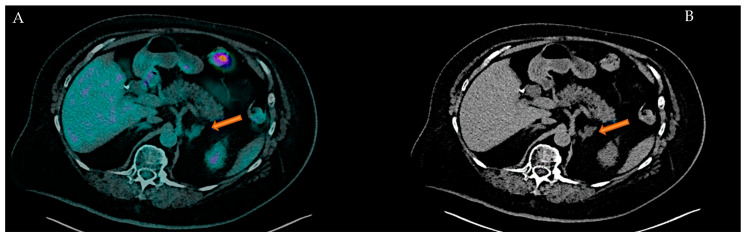
56-year-old male with history of non-small cell lung cancer (NSCLC) and a left adrenal non-secreting-mass indicated by arrows. (**A**): Axial [^18^F]FDG PET/CT fusion imaging shows a focal FDG uptake in left adrenal gland (SUV max: 2.26); (**B**): Axial CT low-dose imaging shows a low-density (UH −15) nodular lesion (maximum transverse diameter: 8 mm) in the left adrenal gland.

Tumor types and their prevalence in this population are reported in Table 1. Breast cancer was the most prevalent cancer type (28.4%) observed in our population; we report 20.0% of secreting adrenal mass (Figure 1), followed by kidney cancer (14.8%), thyroid cancer (12.5%) and the others (Figure 2).

**Figure 3:**
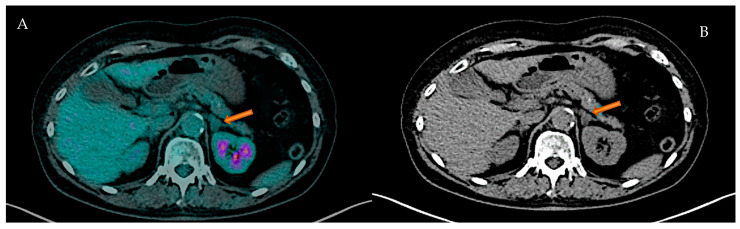
59-year-old female with history of NHL with a left adrenal-secreting mass indicated by arrows. (**A**): Axial [^18^F]FDG PET/CT fusion imaging shows a focal FDG uptake in left adrenal gland (SUV max: 2.13); (**B**): Axial CT low-dose imaging shows a low-density (UH −9.13) nodular lesion (maximum transverse diameter: 11 mm) in the left adrenal gland.

A low rate of adrenal masses (2/88 2.3%) was found in patients with NHL, both studied by CT and [^18^F]FDG PET/CT. One showed biochemical findings of a cortisol secreting adrenal mass (Figure 3).

In this study, most of lesions were bilateral (50.0%); a right lesion was found in 30.7% of patients and a left lesion in 19.3% (Table 1).

## 4. Discussion

Lesions of the adrenal gland frequently occur during radiological examinations for diagnostic and follow-up purposes. First of all, our findings show more than 50% of bilateral adenomas, as described in the literature [1,3]. The correct characterization of adrenal masses is crucial, especially in oncologic patients, given the possibility of many cancer types spreading on the adrenal gland. In our study, the benign nature of the adrenal lesions was based on the CT features, as described above, and SUVmax values < 3 and subsequently confirmed at the 5-year follow-up. Even in the case of benign lesions, such as adenomas, the hormonal status must be assessed, mainly in order to perform an endocrinological diagnosis, mainly because clinical manifestations, due to hormone excesses, may have a negative impact on the quality of life, especially in the oncology setting. Indeed, a secreting lesion requires additional treatments and appropriate clinical management [9,10,11,12,13,14]. Van Doesburg et al. report that the oncological impact of adrenal masses in patients with esophageal cancer is significant, and the detection of a secreting adrenal lesion changed the treatment schedule in terms of both curative and palliative therapies [28].

Functioning adrenal adenomas are characterized by the abnormal production of adrenal steroids, mainly cortisol and aldosterone [6,8].

DST-1 mg is strongly recommended to exclude or confirm the possibility of an autonomous cortisol secretion. Similarly, plasmatic or urinary metanephrines and the assessment of the plasma aldosterone/renin activity ratio are crucial to rule out the presence of pheochromocytoma and primary aldosteronism, respectively [12].

In our study, we aimed to report the prevalence of different cancer types in oncologic patients with known adrenal masses, and we assessed the biochemical profile of these adrenal masses.

In our analysis, 39.8% of lesions were CS, demonstrating a high prevalence of cortisol-secreting lesions in oncological patients, in agreement with many studies that reported a mild autonomous cortisol secretion (MACS) in 43% to 45% of all adrenal tumors in patients without clinically apparent hormone excess [29,30,31].

The failure to suppress cortisol at DST-1 mg in 39.8% of our patients strongly supports the adenomatous nature of the adrenal mass.

The excessive production of cortisol can lead to the development of hypertension, type 2 diabetes mellitus, and obesity [32]. Moreover, MACS has been associated with increased morbidity and mortality [31,33,34,35] and an increase in cardiometabolic risk [36]. These aspects should be considered in the management of cancer patients who undergo anti-tumor therapies. Considering the high prevalence of MACS in our patients, the hormonal status should be always performed in oncological patients in order to improve their survival [37].

Indeed, cortisol regulates many physiological functions including inflammatory and immune responses, as well as stress responses and metabolic processes [38]. However, the association of cancer with a source of cortisol is still a much debated topic. Some studies suggest that cortisol, through its immunomodulatory action, promotes neoplastic progression in certain types of tumors. It is conceivable that increased steroidogenesis may directly or indirectly promote this process [39,40].

Some mechanisms have been evocated to explain it. In some hormone-dependent tumors, such as in the breast, endometrium, and prostate, cortisol-reducing sex hormone-binding globulins induce an increase in sex-free hormones, potentially promoting the development of cancer [41].

Patrova et al., in a recently published retrospective study on 17726 patients, reported a higher incidence of cancer in patients with non-secreting adenomas compared to the controls; this incidence was minimally higher in female individuals than males, thus suggesting a long-term follow-up of adrenal adenomas. They concluded that patients with non-secreting adrenal tumors have a higher risk of developing cancer compared to control subjects without adrenal adenomas [37]; our study, in accordance with Patrova’s findings, does not show a cancer-type related risk to the development of a secreting adenoma.

This may be inconsistent with the involvement of cortisol in immune regulatory mechanisms. Increasing evidence suggests that even so-called “non-secreting” adrenal lesions exhibit intrinsic steroidogenic activity [30,37]; this could then provide an explanation as to how a higher steroidogenic milieu might affect or favor cell proliferation.

This study has some limitations: first, the retrospective and single center design of the study. Despite the large number of patients recruited, the subdivision according to cancer type resulted in a relatively small number of patients per cancer type, thus not allowing for a powerful comparison between groups. Another limitation is the heterogeneous cancer population. Our future aim is to extend the study population and analyze the correlation between SUVs and the TC density with biochemical markers of secreting and non-secreting lesions.

## 5. Conclusions

Contrary to what was previously believed, growing data support the evidence that an adrenal lesion is not uncommon in patients with a positive history of cancer; furthermore, our study demonstrated that it does not necessarily relate to the original cancer [37,42,43]. This retrospective study calculates the prevalence of different types of cancers associated with adrenal lesions and is the first study that indicates the prevalence of cortisol-secreting lesions in an oncologic population.

In our concern, a complete biochemical assessment to identify cortisol-secreting lesions should be considered when a benign adrenal lesion is found to be an “incidentaloma”. Even more, we also strongly believe that, as shown in our study, due to the high prevalence of cortisol-secreting masses, a biochemical characterization of adrenal lesions is crucial in oncological patients for correct clinical and therapeutic management.

## Figures and Tables

**Table 1:** Prevalence of different cancer types in patients with adrenal masses.

Cancer Type *n* (% of Total)	Cortisol Secreting Lesions (CS) *n* (% of Cancer Type)	Non-Secreting Lesions (NS) *n* (% of Cancer Type)	Right Side *n* (%)	Localization Bilateral *n* (%)	Left Side *n* (%)
Breast 25 (28.4)	5 (20.0)	20 (80.0)	5 (20.0)	17 (68.0)	3 (12.0)
Kidney 13 (14.8)	2 (15.4)	11 (84.6)	4 (30.8)	6 (46.1)	3 (23.1)
Thyroid 11 (12.5)	7 (63.6)	4 (36.4)	5 (45.0)	4 (36.0)	2 (19.0)
Lung 8 (9.1)	4 (50.0)	4 (50.0)	3 (37.5)	2 (25.0)	3 (37.5)
Melanoma 7 (7.9)	3 (42.9)	4 (57.1)	5 (71.0)	1 (14.0)	1 (14.0)
Prostate 5 (5.7)	3 (60.0)	2 (40.0)	2 (40.0)	3 (60.0)	0 (0.0)
Colorectal 4 (4.5)	1 (25.0)	3 (75.0)	1 (25.0)	2 (50.0)	1 (25.0)
Bladder 4 (4.5)	2 (50.0)	2 (50.0)	1 (25.0)	3 (75.0)	0 (0.0)
Uterus 3 (3.4)	1 (33.3)	2 (66.7)	1 (33.0)	2 (64.0)	0 (0.0)
Non-Hodgkin lymphoma 2 (2.3)	1 (50.0)	1 (50.0)	0 (0.0)	0 (0.0)	2 (100)
Stomach 2 (2.3)	1 (50.0)	1 (50.0)	0 (0.0)	2 (100)	0 (0.0)
Cervical cancer 1 (1.1)	1 (100)	0 (0.0)	0 (0.0)	1 (100)	0 (0.0)
Laringeal 1 (1.1)	1 (100)	0 (0.0)	0 (0.0)	0 (0.0)	1 (100)
Pancreas 1 (1.1)	1 (100)	0 (0.0)	0 (0.0)	1 (100)	0 (0.0)
Maxillary sinus	1 (100)	0 (0.0)	0 (0.0)	0 (0.0)	1 (100)

## Data Availability

The original data presented in the study are openly available in a publicly accessible repository.

## Abbreviations

CT	computed tomography
MR	magnetic resonance
[^18^F]FDG PET/CT	^18^fluorine-fluorodeoxyglucose positron emission tomography/computed tomography
HU	Hounsfield units
SUV	standardized uptake value
DST	dexamethasone suppression test
CS	cortisol secreting lesions
NS	non-secreting lesions
MACS	mild autonomous cortisol secretion
